# Time series data on typhoid fever incidence during outbreaks from 2000 to 2022

**DOI:** 10.1038/s41597-024-04289-7

**Published:** 2025-01-16

**Authors:** Dae-Hyup Koh, Monica Duong, Nodar Kipshidze, Virginia E. Pitzer, Jong-Hoon Kim

**Affiliations:** 1 Epidemiology, Public Health, Impact, International Vaccine Institute, Seoul, Korea; 2 Graduate School of Public Health, Yonsei University, Seoul, Korea; 3 Dalla Lana School of Public Health, University of Toronto, Ontario, Canada; 4 Department of Epidemiology of Microbial Diseases, Yale School of Public Health, New Haven, CT USA

**Keywords:** Bacterial infection, Intestinal diseases

## Abstract

This article presents a comprehensive dataset compiling reported cases of typhoid fever from culture-confirmed outbreaks across various geographical locations from 2000 through 2022, categorized into daily, weekly, and monthly time series. The dataset was curated by identifying peer-reviewed epidemiological studies available in PubMed, OVID-Medline, and OVID-Embase. Time-series incidence data were extracted from plots using WebPlotDigitizer, followed by verification of a subset of the dataset. The primary aim of this dataset is to serve as a foundational tool for researchers and policymakers, enabling the development of robust, model-based strategies for the control of typhoid fever outbreaks. The article describes the method by which the dataset has been compiled and how the quality of the data has been verified. Furthermore, it discusses the dataset’s potential applications in optimizing vaccination campaigns, improving public health planning, and tailoring interventions to specific epidemiologic contexts. This article contributes significantly to the field of infectious disease modeling, offering a valuable resource for enhancing typhoid fever control measures globally.

## Background & Summary

Typhoid fever, caused by the bacterium *Salmonella enterica* serovar Typhi (*S*. Typhi)^1^, remains a significant public health challenge in many parts of the world, particularly in low- and middle-income countries (LMICs) where access to clean water and sanitation is limited. While estimates vary, typhoid fever is believed to cause at least 10.9 million (95% uncertainty interval [UI]: 9.3–12.6) cases and 116.8 thousand (95% UI: 65.4–187.7) deaths globally in 2017^2–5^.

Treatment options for typhoid include first-line antibiotics like chloramphenicol, ampicillin, and trimethoprim-sulfamethoxazole, as well as fluoroquinolone such as ciprofloxacin. However, antimicrobial resistant *S*. Typhi, including multi-drug resistance to the three first-line antibiotics and fluoroquinolone non-susceptibility, are common in different parts of the world, which emerged long before 2016^6,7^. Furthermore, extensively-drug resistant typhoid has recently emerged, which is, in addition to the aforementioned antibiotics, also resistant to third-generation cephalosporins such as ceftriaxone and was first reported in Pakistan in 2016^8^, leaving only azithromycin as oral antibiotic treatment^9^.

Public health interventions to reduce typhoid burden include a combination of community outreach initiatives, education campaigns, improvements in water, sanitation, and hygiene, and vaccination. Recent meta-analyses show consistently reduced odds of typhoid fever in individuals with access to improved hygiene, improved water sources, and treated water^10^. Three typhoid conjugate vaccines (TCV) have been prequalified by the World Health Organization (WHO) as of February 2024^11,12^. These vaccines showed 83% (95% CI, 77–87%)^13–17^ after two years of vaccination, and WHO advises prioritizing TCV implementation in nations with the highest rates of typhoid and/or antimicrobial-resistant *S*. Typhi^18^.

Despite advances in vaccination and treatment strategies, typhoid fever continues to affect millions annually, leading to substantial morbidity and mortality, and there continue to be large-scale outbreaks of typhoid fever^19^. The dynamic nature of typhoid fever transmission, driven by the interaction of environmental, social, and host- and pathogen-associated factors, necessitates statistical and mathematical models to predict outbreaks, evaluate intervention strategies, and inform public health policy and decision-making. The availability of robust and comprehensive data sets, especially time series data reflecting the incidence and spread of typhoid fever over time, along with contextual information including the response to the outbreak, is crucial for the development of accurate and reliable disease models. However, the compilation and synthesis of such data have been fragmented, with relevant time series data scattered across various peer-reviewed articles, often in formats not readily amenable to aggregation or analysis.

To address this gap, we conducted a systematic review to extract, standardize, and compile time series data on typhoid outbreaks. We also collected contextual information, such as geographical location, diagnostic methods used, history of typhoid transmission in the community (to infer existing population immunity), presence and type of antimicrobial resistance, and any interventions implemented during the outbreak. The documented outbreaks may not fully represent the true incidence or spatiotemporal distribution of typhoid fever epidemics as surveillance systems in LMICs are often inadequate, and even gold-standard diagnostics have suboptimal sensitivity^20^. However, our goal was to develop a comprehensive dataset capturing the temporal dynamics of typhoid fever incidence during outbreaks across various geographical locations and time periods, providing a valuable resource for researchers.

## Methods

### Study design

We conducted a systematic review pursuant to the Cochrane Handbook and the Preferred Reporting Items for Systematic Review and Meta-Analysis (PRISMA) statement^21^. The protocol for this review was registered in the International Prospective Register of Systematic Reviews (PROSPERO) (registration number: CRD42024465039).

### Search strategy

We used a variety of search terms that capture differences in characterizing and describing an outbreak of typhoid fever to identify relevant literature from PubMed, OVID-Medline, and OVID-Embase. We restricted our search to studies published between January 1, 2000, and July 31, 2023, to focus on recent outbreaks, which would be more relevant for evaluating interventions against future outbreaks. We restricted our search to studies written in English. We excluded pre-prints and ongoing studies and supplemented our search by examining references cited in relevant publications. The complete search terms for each database are detailed in Table 1.

**Table 1:** Search strings used in the systematic review.

Database	Search Query
Pubmed including OVID-Medline	(((“typhoid fever”[MeSH Terms] OR “salmonella typhi”[MeSH Terms] OR (((“s typhi”[Text Word] OR “salmonella typhi”[Text Word] OR “salmonella enterica serovar* typhi”[Title/Abstract]) AND “s typhi”[Title/Abstract]) OR “salmonella typhi”[Title/Abstract] OR “salmonella enterica serovar* typhi”[Title/Abstract]) OR (“typhoid”[Text Word] OR “enteric fever”[Text Word])) AND (“disease outbreaks”[MeSH Terms] OR “epidemics”[MeSH Terms] OR “outbreak*”[Text Word])) NOT (“animals”[MeSH Terms] NOT “Humans”[MeSH Terms])) AND 2000/01/01:2023/07/31[Date - Publication]
OVID-Embase	(((‘typhoid fever’/exp OR (‘typhoid’:ti,ab,kw OR ‘enteric fever’:ti,ab,kw) OR ‘salmonella enterica serovar typhi’/exp OR (‘s. typhi’:ti,ab,kw OR ‘salmonella typhi’:ti,ab,kw OR ‘salmonella enterica serovar* typhi’:ti,ab,kw)) AND (‘epidemic’/exp OR ‘outbreak*‘:ti,ab,kw)) NOT (((‘animal’/exp OR animal) AND ‘experiment’/exp OR ‘nonhuman’/exp) NOT ((exp AND ‘human’/exp OR human) AND ‘experiment’/exp))) AND [01-01-2000]/sd NOT [31-07-2023]/sd

### Eligibility criteria and study selection

We evaluated the eligibility of studies by examining the following specific aspects of the study: diagnostic method and availability of daily, weekly, or monthly incidence. We included original reports of typhoid fever outbreaks, published between 1st January 2000 to 1st July 2023, in which *S*. Typhi was identified through blood or bone marrow culture at least in a subset of the reported typhoid cases.

In the case of studies without epidemic curves or a table of incidence, we reached out to the authors of the original articles requesting time series data. We excluded studies from which time series data could not be extracted.

Two reviewers (D.H.K. and M.D.) assessed the studies for inclusion after screening the title and abstract independently.

### Data extraction & synthesis

**Fig. 1:**
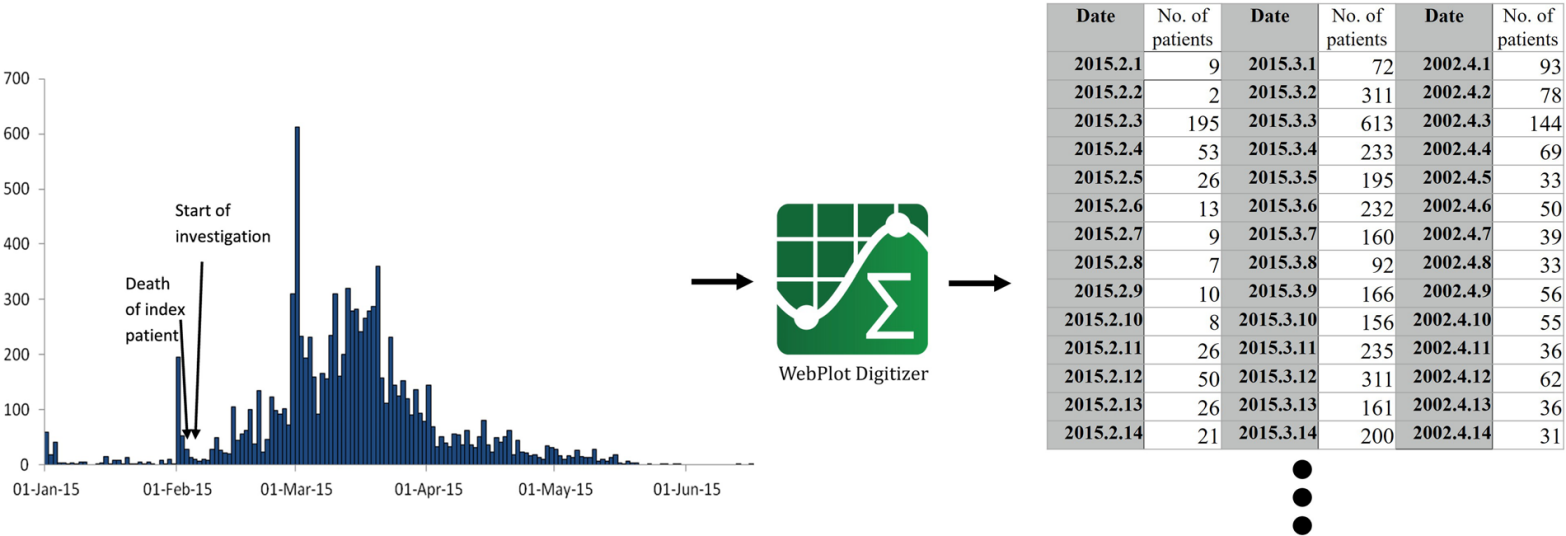
Extraction of daily incidence of typhoid fever cases during the outbreak using WebPlotDigitizer.

All the data were extracted from published articles via Covidence^22^. Definitions for the case and the outbreak of typhoid fever were based on those by the study authors. Time series of incidence of typhoid fever were extracted from the plot of the epidemic curve using WebPlotDigitizer^23,24^, software that extracts numerical values from the location of data points relative to their location on the X and Y axes (Fig. 1). Variables such as the year and geographical location of the typhoid outbreak, reporting year, start and end dates, duration, diagnostic methods, intervention methods, reporting frequency, total suspected/confirmed/probable cases, attack rate, total deaths, case fatality ratio (CFR), and antimicrobial resistance patterns were retrieved. Population and outbreak area details were extracted if mentioned in the article. Extracted data were compiled into a separate Excel sheet. Disagreements between the two reviewers were resolved after discussions with the last author (J.-H. K.).

We did not merge studies even if some of the studies report the same outbreaks. Studies report data at different time resolutions, which may be of use for different purposes. For example, Imanishi *et al*.^25^, Polonsky *et al*.^26^, and Muti *et al*.^27^ all report outbreaks at Harare, Zimbabwe but they report the incidence at monthly, weekly, and daily time scales, respectively, while Imanishi *et al*. encapsulates the other two data. Similarly, Qamar *et al*.^28^ and Yousafzai *et al*.^29^ studies report weekly typhoid incidence during outbreaks in Hyderabad, Pakistan. Again, we did not merge these two studies as these studies differ in that Qamar *et al*. and Yousafzai *et al*. report different cut-off dates of weekly incidence.

### Included dataset

**Fig. 2:**
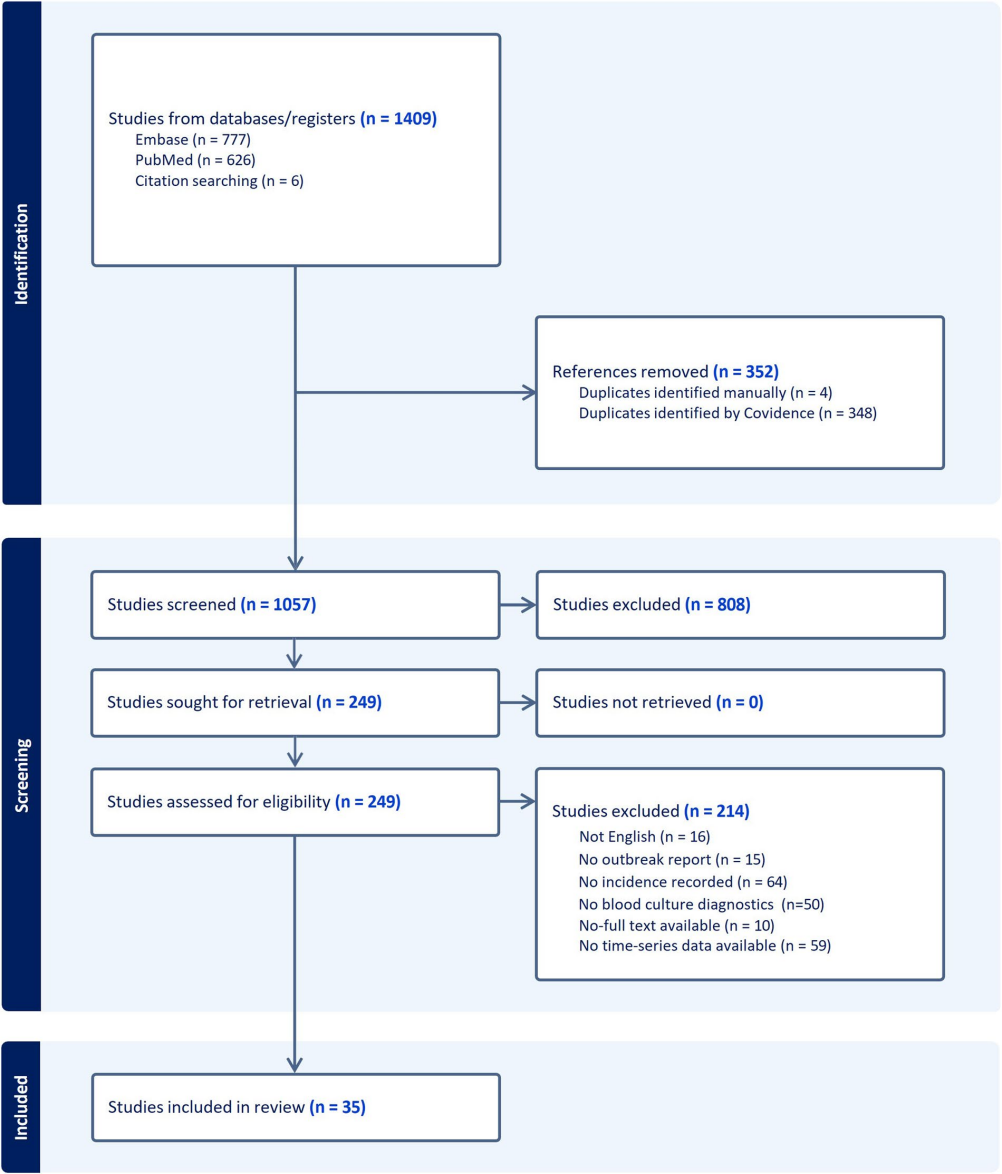
Preferred reporting items for systematic reviews and meta-analyses (PRISMA) study flowchart.

A total of 1,409 articles were retrieved from the online databases, resulting in 1,057 articles after excluding duplicates. Following the selection criteria, 249 studies were selected for full-text review. Finally, we identified 35 unique peer-reviewed articles that met our inclusion criteria^25–59^, which resulted in 39 typhoid outbreaks in which daily, weekly, or monthly incidences of culture-confirmed typhoid fever were available (Fig. 2, Table 2).

**Table 2:** Summary of included studies with available links and copyright credits.

First author	Publication Year	Article Title	Published Journal	Credit to the data source or Copyright clearance	DOI or full-text source	Reference Number
Aye	2004	Typhoid fever outbreak in Madaya Township, Mandalay Division, Myanmar, September 2000	J Med Assoc Thai	Open Access	https://medassocthai.org/journal/files/Vol87_No4_395.pdf	^32^
Michel	2005	Outbreak of typhoid fever in vaccinated members of the French Armed Forces in the Ivory Coast	Eur J Epidemiol	Data from Fig. 1. Michel, R., Garnotel, E., Spiegel, A., Morillon, M., Saliou, P., & Boutin, J. P., Outbreak of typhoid fever in vaccinated members of the French Armed Forces in the Ivory Coast, European journal of epidemiology, 20, 635-642., Copyright Springer Nature (2005).	10.1007/s10654-005-7454-6	^48^
Lewis	2005	Typhoid fever: a massive, single-point source, multidrug-resistant outbreak in Nepal	Clin Infect Dis	Data from Fig. 2. Lewis, M. D., Serichantalergs, O., Pitarangsi, C., Chuanak, N., Mason, C. J., Regmi, L. R.,… & Malla, S., Typhoid fever: a massive, single-point source, multidrug-resistant outbreak in Nepal. Clinical Infectious Diseases, 40(4), 554-561., Copyright Oxford University Press (2005).	10.1086/427503	^44^
Al-Sanouri	2008	Emergence of plasmid-mediated multidrug resistance in epidemic and non-epidemic strains of Salmonella enterica serotype Typhi from Jordan	J Infect Dev Ctries	Open Access	10.3855/jidc.225	^30^
Muehlen	2007	Outbreak of domestically acquired typhoid fever in Leipzig, Germany, June 2004	Eurosurveillance	Open Access	10.2807/ESM.12.02.00684-EN	^49^
Muyembe-Tamfum	2009	An outbreak of peritonitis caused by multidrug-resistant Salmonella Typhi in Kinshasa, Democratic Republic of Congo	Travel Med Infect Dis	**Data from** Fig. 1**. Muyembe-Tamfum, J. J., J. Veyi, M. Kaswa, O. Lunguya, J. Verhaegen, and M. Boelaert., An outbreak of peritonitis caused by multidrug-resistant Salmonella Typhi in Kinshasa, Democratic Republic of Congo, Travel medicine and infectious disease, 7(1), 40-43., Copyright Elsevier (2009)**.	10.1016/j.tmaid.2008.12.006	^50^
Holt	2011	Temporal fluctuation of multidrug resistant salmonella typhi haplotypes in the mekong river delta region of Vietnam	PLoS Negl Trop Dis	Open Access	10.1371/journal.pntd.0000929	^40^
Keddy	2011	Molecular epidemiological investigation of a typhoid fever outbreak in South Africa, 2005: the relationship to a previous epidemic in 1993	Epidemiol Infect	Data from Fig. 1. Keddy, K. H., Sooka, A., Ismail, H., Smith, A. M., Weber, I., Letsoalo, M. E., & Harris, B. N., Molecular epidemiological investigation of a typhoid fever outbreak in South Africa, 2005: the relationship to a previous epidemic in 1993. Epidemiology & Infection, 139(8), 1239-1245., Copyright Cambridge University Press (2011).	10.1017/s0950268810002207	^43^
Neil	2012	A large outbreak of typhoid fever associated with a high rate of intestinal perforation in Kasese District, Uganda, 2008-2009	Clin Infect Dis	Open Access	10.1093/cid/cis025	^52^
Bayram	2011	Epidemiological characteristics and molecular typing of Salmonella enterica serovar Typhi during a waterborne outbreak in Eastern Anatolia	Ann Trop Med Parasitol	Data from Fig. 2. Bayram, Y., Güdücüoğlu, H., Otlu, B., Aypak, C., Gürsoy, N. C., Uluç, H., & Berktaş, M. Epidemiological characteristics and molecular typing of Salmonella enterica serovar Typhi during a waterborne outbreak in Eastern Anatolia. Annals of Tropical Medicine & Parasitology, 105(5), 359-365., Copyright Taylor & Francis (2011).	10.1179/1364859411y.0000000024	^34^
Scobie	2014	Impact of a targeted typhoid vaccination campaign following cyclone Tomas, Republic of Fiji, 2010	Am J Trop Med Hyg	Data from Fig. 2. Scobie, H. M., Nilles, E., Kama, M., Kool, J. L., Mintz, E., Wannemuehler, K. A.,… & Date, K. Impact of a targeted typhoid vaccination campaign following cyclone Tomas, Republic of Fiji, 2010. The American journal of tropical medicine and hygiene, 90(6), 1031., Copyright ASTMH (2014).	10.4269/ajtmh.13-0728	^56^
Lutterloh	2012	Multidrug-resistant typhoid fever with neurologic findings on the Malawi-Mozambique border	Clin Infect Dis	Open Access	10.1093/cid/cis012	^46^
Limpitikul	2014	Typhoid outbreak in Songkhla, Thailand 2009-2011: clinical outcomes, susceptibility patterns, and reliability of serology tests	PLoS One	Open Access	10.1371/journal.pone.0111768	^45^
Hendriksen	2015	Genomic signature of multidrug-resistant Salmonella enterica serovar typhi isolates related to a massive outbreak in Zambia between 2010 and 2012	J Clin Microbiol	Data from Fig. 1. Hendriksen, R. S., Leekitcharoenphon, P., Lukjancenko, O., Lukwesa-Musyani, C., Tambatamba, B., Mwaba, J.,… & Mwansa, J. C., Genomic signature of multidrug-resistant Salmonella enterica serovar Typhi isolates related to a massive outbreak in Zambia between 2010 and 2012., Journal of clinical microbiology, 53(1), 262-272., Copyright ASM (2015).	10.1128/jcm.02026-14	^39^
Walters	2014	Shifts in geographic distribution and antimicrobial resistance during a prolonged typhoid fever outbreak–Bundibugyo and Kasese Districts, Uganda, 2009-2011	PLoS Negl Trop Dis	Open Access	10.1371/journal.pntd.0002726	^58^
Ali	2017	Localised transmission hotspots of a typhoid fever outbreak in the Democratic Republic of Congo	Pan Afr Med J	Open Access	10.11604/pamj.2017.28.179.10208	^31^
Polonsky	2014	Descriptive epidemiology of typhoid fever during an epidemic in Harare, Zimbabwe, 2012	PLoS One	Open Access	10.1371/journal.pone.0114702	^26^
Muti	2014	Typhoid outbreak investigation in Dzivaresekwa, suburb of Harare City, Zimbabwe, 2011	Pan Afr Med J	Open Access	10.11604/pamj.2014.18.309.4288	^27^
Imanishi	2014	Household water treatment uptake during a public health response to a large typhoid fever outbreak in Harare, Zimbabwe	Am J Trop Med Hyg	Data from Fig. 2. Imanishi, M., Kweza, P. F., Slayton, R. B., Urayai, T., Ziro, O., Mushayi, W.,… & Zimbabwe Typhoid Fever Outbreak Working Group. Household water treatment uptake during a public health response to a large typhoid fever outbreak in Harare, Zimbabwe. The American journal of tropical medicine and hygiene, 90(5), 945., Copyright ASTMH (2014).	10.4269/ajtmh.13-0497	^25^
Cherian	2015	An outbreak investigation of typhoid fever in Pondicherry, South India, 2013	Int J Med Sci Public Health	Open Access	http://journalarticle.ukm.my/8779/1/P.256-261.pdf	^36^
Roy	2016	Epidemiological investigation of an outbreak of typhoid fever in Jorhat town of Assam, India	Indian J Med Res	Open Access	https://pmc.ncbi.nlm.nih.gov/articles/PMC5345307/	^55^
Kabwama	2017	A large and persistent outbreak of typhoid fever caused by consuming contaminated water and street-vended beverages: Kampala, Uganda, January - June 2015	BMC Public Health	Open Access	10.1186/s12889-016-4002-0	^42^
Burnsed	2018	Use of whole genome sequencing to complement characterisation of a typhoid fever outbreak among a Marshallese community: Oklahoma, 2015	Epidemiol Infect	Open Access	10.1017/s0950268818002601	^35^
Qamar	2018	Outbreak investigation of ceftriaxone-resistant Salmonella enterica serotype Typhi and its risk factors among the general population in Hyderabad, Pakistan: a matched case-control study	Lancet Infect Dis	Data from Fig. 2. Qamar, F. N., Yousafzai, M. T., Khalid, M., Kazi, A. M., Lohana, H., Karim, S.,… & Hasan, R. Outbreak investigation of ceftriaxone-resistant Salmonella enterica serotype Typhi and its risk factors among the general population in Hyderabad, Pakistan: a matched case-control study. The Lancet Infectious Diseases, 18(12), 1368-1376., Copyright Elsevier (2018).	10.1016/s1473-3099(18)30483-3	^28^
Davis	2018	Notes from the Field: Typhoid Fever Outbreak - Harare, Zimbabwe, October 2016-March 2017	MMWR Morb Mortal Wkly Rep	Open Access	10.15585/mmwr.mm6711a7	^37^
Hu	2022	Genomic Investigation Reveals a Community Typhoid Outbreak Caused by Contaminated Drinking Water in China, 2016	Front Med (Lausanne)	Open Access	10.3389/fmed.2022.753085	^41^
Yousafzai	2019	Ceftriaxone-resistant Salmonella Typhi Outbreak in Hyderabad City of Sindh, Pakistan: High Time for the Introduction of Typhoid Conjugate Vaccine	Clin Infect Dis	Open Access	10.1093/cid/ciy877	^29^
Hechaichi	2023	Outbreak Investigation of Typhoid Fever in the District of Gabes, South of Tunisia	Epidemiologia (Basel)	Open Access	10.3390/epidemiologia4030023	^38^
N’Cho	2019	Notes from the Field: Typhoid Fever Outbreak - Harare, Zimbabwe, October 2017-February 2018	MMWR Morb Mortal Wkly Rep	Open Access	10.15585/mmwr.mm6802a5	^51^
Bano-Zaidi	2018	Typhoid fever outbreak with severe complications in Yucatan, Mexico	Lancet Glob Health	Open Access	10.1016/s2214-109x(18)30312-7	^33^
Makungo	2020	Epidemiological investigation of a typhoid fever outbreak in Sekhukhune District, Limpopo province, South Africa - 2017	S Afr J Infect Dis	Open Access	10.4102/sajid.v35i1.107	^47^
Poncin	2022	Implementation of an outbreak response vaccination campaign with typhoid conjugate vaccine - Harare, Zimbabwe, 2019	Vaccine X	Open Access	10.1016/j.jvacx.2022.100201	^54^
Nimonkar	2022	Clinico-epidemiological study of an outbreak of typhoid in North India	J Family Med Prim Care	CC BY-NC-SA	10.4103/jfmpc.jfmpc_2498_21	^53^
Srinivasan	2022	Outbreak of Typhoid Fever in Children of Urban Vellore: A Report from the Surveillance for Enteric Fever in India Cohort	Am J Trop Med Hyg	Open Access	10.4269/ajtmh.21-0593	^57^
Wang	2022	Extensively Drug-Resistant (XDR) Salmonella Typhi Outbreak by Waterborne Infection - Beijing Municipality, China, January-February 2022	China CDC Wkly	CC BY-NC	10.46234/ccdcw2022.062	^59^

## Data Records

All data extracted from this systematic review is recorded in 3 spreadsheets. The dataset is available at Open Science Framework (https://osf.io/n9cke/)^60^ with DOI of 10.17605/OSF.IO/N9CKE, “Typhoid_Outbreak_Time_Series_2000_2022.xlsx”.

Each row of the spreadsheet corresponds to a unique typhoid record. Each column in the sheets represents a variable as described in Table 3 and the “Dictionary” sheet of the online dataset.

**Table 3:** List of variables with description.

Column title	Description
Sheet #1 Typhoid Outbreaks Summary	
Study ID	Study # indicated by first author with published year
Outbreak start year	Typhoid outbreak start year stated in the literature or epidemic curve
Outbreak end year	Typhoid outbreak end year stated in the literature or epidemic curve
Location	Country and district of typhoid outbreak
Country	Country of typhoid outbreak
Country 3-letter ISO	3-letter International Organization for Standardization (ISO) code defined by ISO 3166 https://www.iso.org/obp/ui/#search/code/
Admin0_1	Country of typhoid outbreak
Admin0_2	Country2 of typhoid outbreak
Admin1	District of typhoid outbreak
WHO region	Location of outbreak within six World Health Organization regions
Diagnostic method	Diagnostic method used to differentiate probable or confirmed cases from suspected cases
Diagnostic method_1	Diagnostic test by Blood culture
Diagnostic method_2	Diagnostic test by Bone marrow culture
Diagnostic method_3	Diagnostic test by Faecal culture
Diagnostic method_4	Diagnostic test by Urine culture
Diagnostic method_5	Diagnostic test by Culture (not specified)
Diagnostic method_6	Diagnostic test by Clinical Symptoms
Diagnostic method_7	Diagnostic test by Widal test
Diagnostic method_8	Diagnostic test by TUBEX
Diagnostic method_9	Diagnostic test by etxtra methods
Suspected/Confirmed/Probable	Typhoid incidence case types reported in the article; if the authors reported confirmed cases by Widal test, Probable was added to be consistent with WHO guidelines
AMR status	AMR status reported during the outbreak
Intervention	Any kind of intervention reported during the outbreak
Time unit	Reporting interval of cases whether daily, weekly, or monthly
Start date	Start date of the outbreak reported in the article; if not reported, the date was extracted from time-series figure or epidemic curve
End date	End date of the outbreak reported in the article; if not reported, the date was extracted from a time series figure or epidemic curve
Peak date	Peak date of the outbreak reported in the article; if not reported, the date was extracted from a time series figure or epidemic curve
Intervention date	Intervention date of the outbreak reported in the article; if not reported, the date was extracted from a time series figure or epidemic curve
Case definitions	Suspected, probable, and confirmed cases defined by the author
Total suspected cases	Total number of suspected cases reported by the authors.
Lab tested by blood/bone marrow culture	Total number of suspected cases that were tested by blood or bone marrow culture
Total confirmed cases	Total number of confirmed cases reported by the authors
Confirmed cases by blood/ bone marrow culture	Total number of confirmed cases only by blood or bone marrow culture
Total probable cases	Total number of probable cases reported by the author; if the authors reported confirmed cases by Widal test, it was moved to this column to be consistent with WHO guidelines
Number of hospitalized	Number of hospitalized patients reported by the authors
Number of complications	Number of complications reported by the authors
Total deaths	Total deaths reported by the authors
Attack rate (%)	Attack rate reported by the authors
CFR	Case fatality ratio reported by the authors
Population	Population reported by the authors, or reported population in case the population of the investigated site of outbreak could be searched by Google
Notes	Any notes that need consideration when this datasheet is used
**Sheet #2 Time series data**	
Study ID	Study # indicated by first author with published year
Start date	Start date of the outbreak reported in the article, if not reported, the date was extracted from a time series figure or epidemic curve
End date	End date of the outbreak reported in the article, if not reported, the date was extracted from a time series figure or epidemic curve
No. of Patients	Total number of patients including suspected, confirmed, and probable cases extracted from time-series figure or epidemic curve
Suspected	Total number of suspected cases extracted from time-series figure or epidemic curve
Confirmed	Total number of confirmed cases extracted from time series figure or epidemic curve
Probable	Total number of probable cases extracted from time series figure or epidemic curve
**Sheet #3 Age distribution**	
Study ID	Study # indicated by first author with published year
Location	Country and District of typhoid outbreak
Median age (IQR)	Median age (interquartile range) reported by the authors
Mean age (SD)	Mean age (standard deviation) reported by the authors

## Technical Validation

All the data were extracted from published articles indexed in PubMed, OVID-Medline, and OVID-Embase databases via Covidence. Two independent reviewers (DHK and MD) conducted title-abstract screening and full-text screening according to predefined inclusion criteria. To reduce the error, data extraction was also performed independently by two reviewers (DHK and MD) with separate Excel sheets. Disagreements were resolved through discussion with the last author (JHK). Time series collected from the epidemic curve using WebPlotDigitizer were validated by measuring the absolute difference and the error rate (%) defined as below:

$$\mathrm{Absolute}\,\mathrm{difference}=|\mathrm{extracted}\,\mathrm{case}\,-\,\mathrm{true}\,\mathrm{case}|$$

$$\mathrm{Error}\,\mathrm{rate} \% =\frac{|\mathrm{extracted}\,\mathrm{case}-\mathrm{true}\,\mathrm{case}|}{\mathrm{true}\,\mathrm{case}}\times 100$$

Here, true cases represent the number of typhoid cases shared by the authors. Seven datasets were received by the authors of published outbreak articles and compared against the extracted datasets. The average absolute difference and the error rate were 1.4 cases and 0.2%, respectively.

We extracted data for the age distribution analysis from figures in published articles using WebPlotDigitizer for four studies^28,39,46,58^ where age distribution was only reported by figure.

## Supplementary information

Supplementary Information

## Data Availability

No custom code was used in extracting the data or analysis in this manuscript.
