# Supplementary material for: Time series data on typhoid fever incidence during outbreaks from 2000 to 2022
Source: Sci Data. 2025 Jan 16;12:94. doi: 10.1038/s41597-024-04289-7 (PMC11739417; doi:10.1038/s41597-024-04289-7)
Supplement: Supplementary file 1 — Supplementary Information [file 41597_2024_4289_MOESM1_ESM.docx]

### Supplementary Results

Among the 39 documented outbreaks, we identified duplicate reports from Hyderabad, Pakistan, and Harare, Zimbabwe in different journals with overlapping outbreak periods^1-35^. We selected the studies that covered the entire reported duration and excluded those with shorter timeframes. Specifically, we chose Qamar et al.^29^ over Yousafzai et al.^35^ for Hyderabad, Pakistan, and Imanishi et al.^13^ over Polonsky et al.^27^, which reports two outbreaks, and Muti et al.^22^ for Harare, Zimbabwe. This resulted in 35 unique typhoid outbreaks across 21 countries, totaling 36,014 suspected cases.

Data were stratified by WHO Region. In the African Region, there were 16 outbreaks, followed by 9 outbreaks in Southeast Asia Region. The African Region reported the highest median number of suspected cases: 784 (range: 10 - 10,230), followed by European Region with 322 cases (range: 6 - 637). Of all suspected cases, 3,880 (10.8%) were confirmed through culture of blood, bone marrow, or stool. The Eastern Mediterranean region had the highest median number of confirmed cases: 74 (range: 24 - 486). The median duration of outbreaks across all regions was 19 weeks (range: 1 - 209). The median time to the peak of the outbreak was 6 weeks (range: 1 - 122 weeks) (Supplementary Table 1).


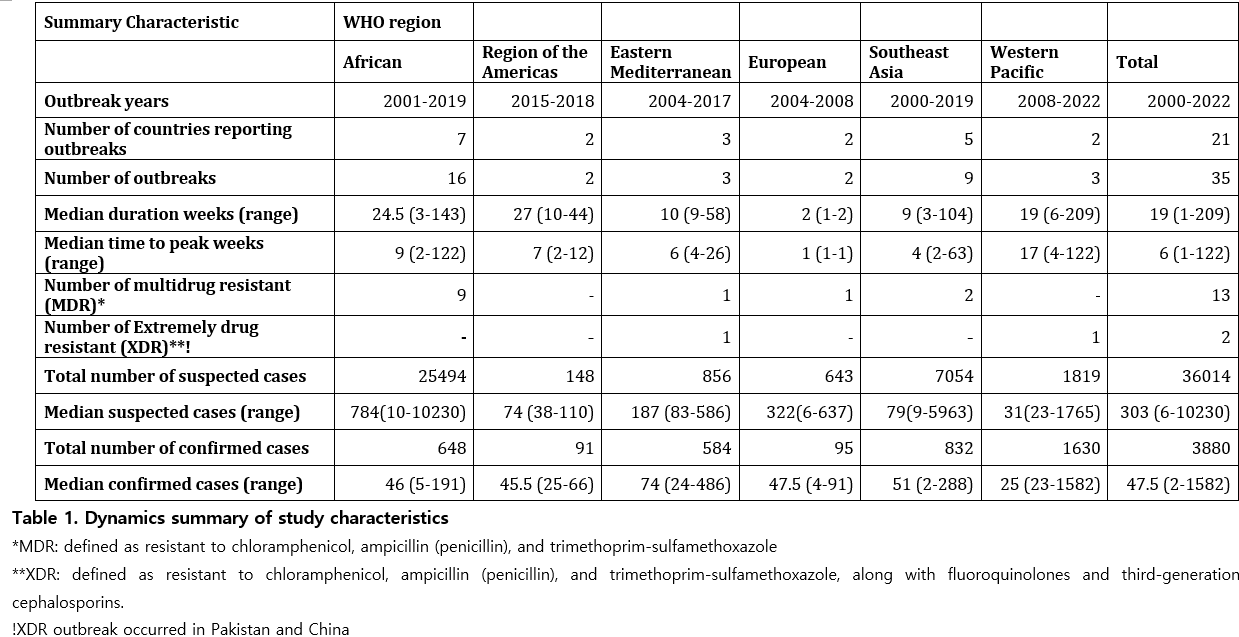


Supplementary Table 1. Summary of study characteristics

* MDR: Multi-drug resistant, defined as resistant to chloramphenicol, ampicillin (penicillin), and trimethoprim-sulfamethoxazole

** XDR: Extensively drug resistant, defined as resistant to chloramphenicol, ampicillin (penicillin), and trimethoprim-sulfamethoxazole, along with fluoroquinolones and third-generation cephalosporins.

! XDR outbreaks were reported in Pakistan and China

Out of 25 typhoid outbreaks for which antimicrobial resistance (AMR) of the *Salmonella enterica* serovar Typhi (*S*. Typhi) isolates were tested, 21 (84%) were associated with AMR. Specifically, out of which 13 and 2 outbreaks were associated with MDR and XDR *S*. Typhi, respectively. The two XDR *S*. Typhi outbreaks occurred in Pakistan (2016)^29^ and China (2022)^34^.

When evaluating the occurrence of outbreaks by country, the country with the highest occurrence of reported typhoid outbreaks were Zimbabwe, Uganda and India with 4 reported outbreaks. (Supplementary Figure 1)


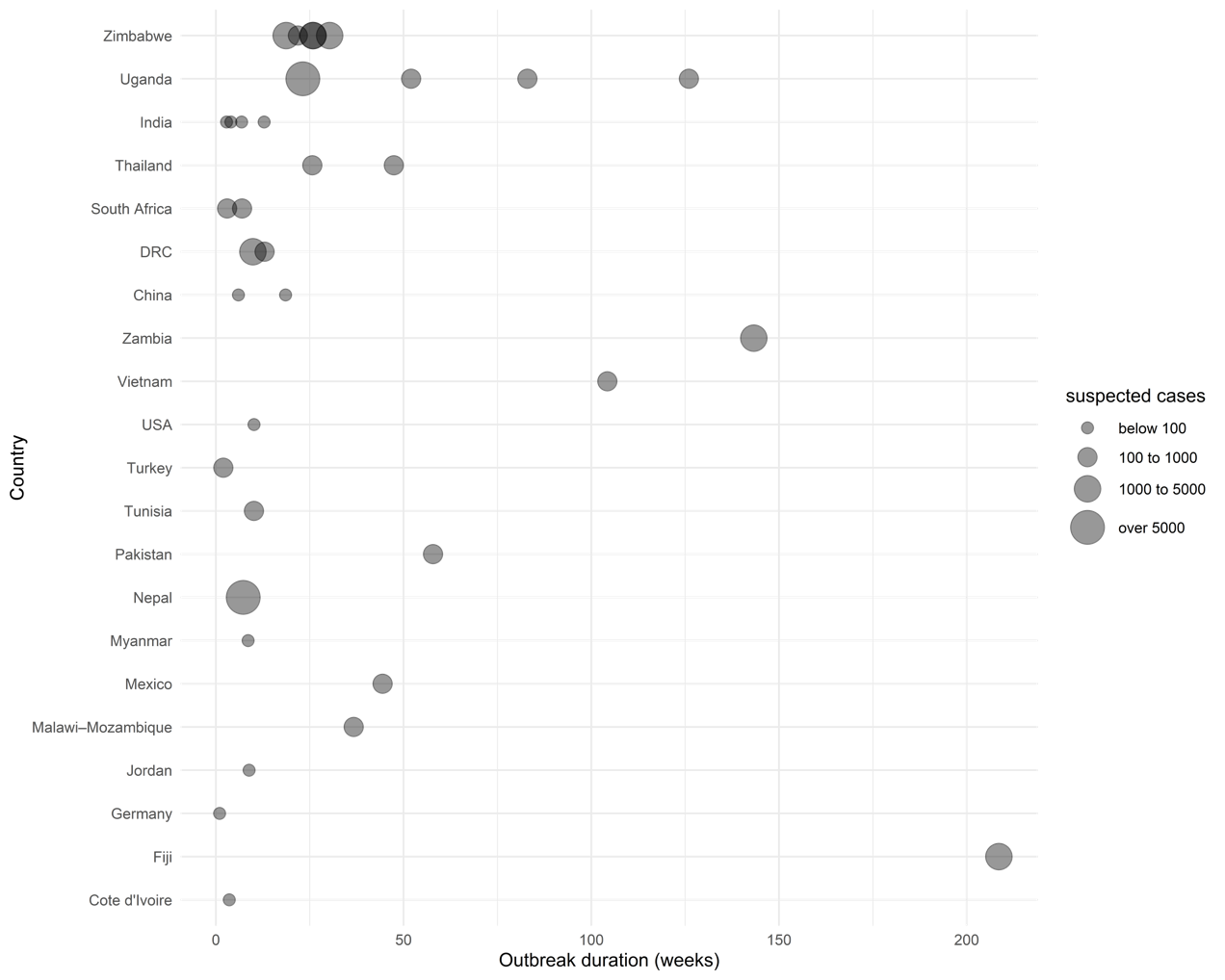


Supplementary Figure 1. Outbreak duration (in weeks) by country. Out of the four outbreaks that occurred in Zimbabwe, one outbreak was divided into the two subdistricts of Harare: Dzivareskewa and Kuwadzana. Outbreak duration indicates how long the time series is accounting for the unit time .

Next, we assessed the age distribution of cases across outbreaks. Age group-specific incidences were reported for 11 outbreaks reporting 6,475 suspected or culture-confirmed typhoid cases. Children younger than 14 years of age comprised over half of all cases. The median proportion of children aged 5-14 years was 36.4% (IQR 31.2% - 55.4%), for children under the 5 years of age it was 14.1% (IQR 11.1% - 22.4%), and for those aged 15 years and older it was 47.5% (IQR 18.0% - 55.8%) (Supplementary Figure 2). Out of the 11 outbreaks, 3 reported the age distribution based solely on culture-confirmed cases, with a total of 544 patients. (Supplementary Figure 3).


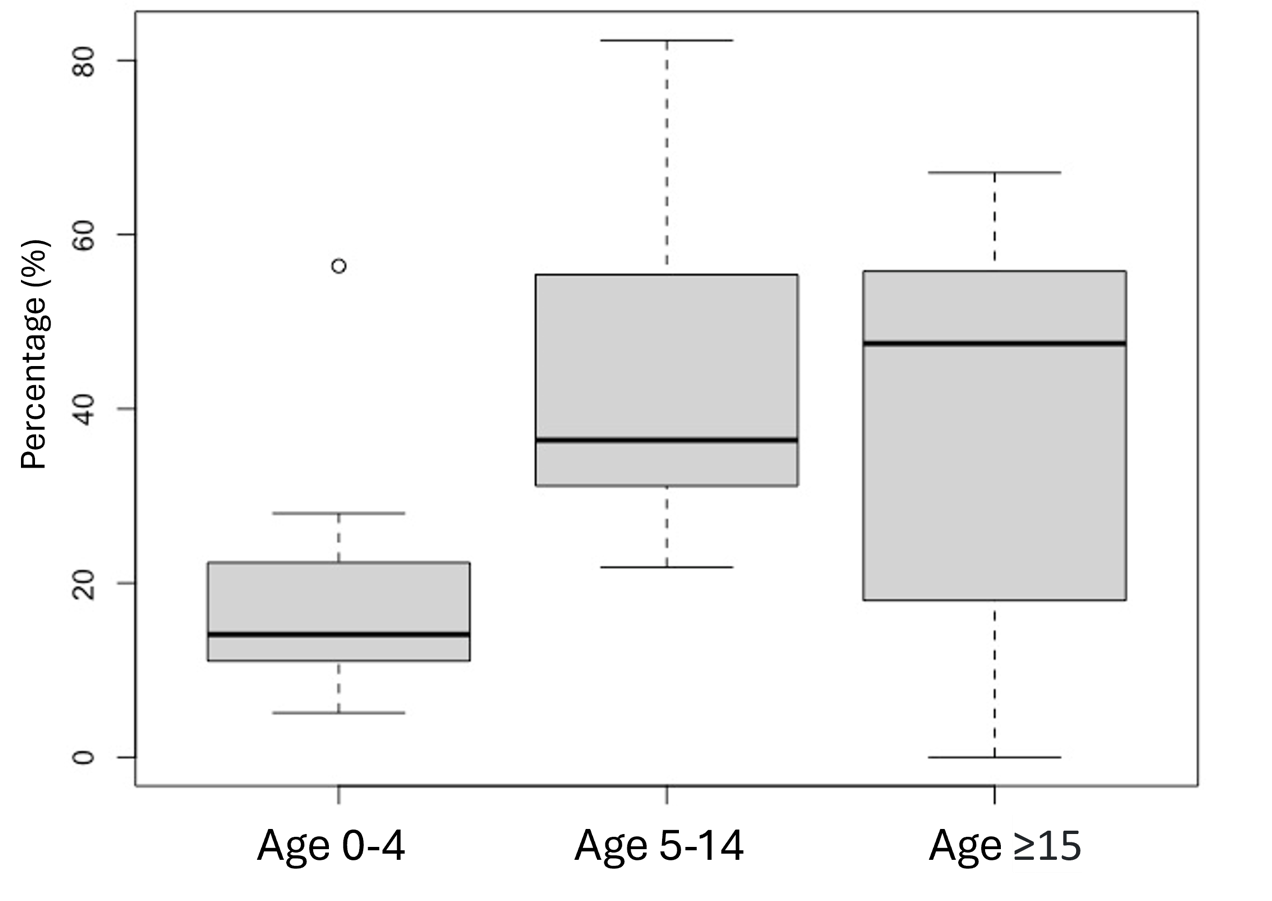

Supplementary Figure 2. Age distribution of cases among typhoid outbreaks. Boxes, horizontal black lines, whiskers, and dots represent IQR (interquartile range), median, 1.5 IQR, and outliers, respectively.


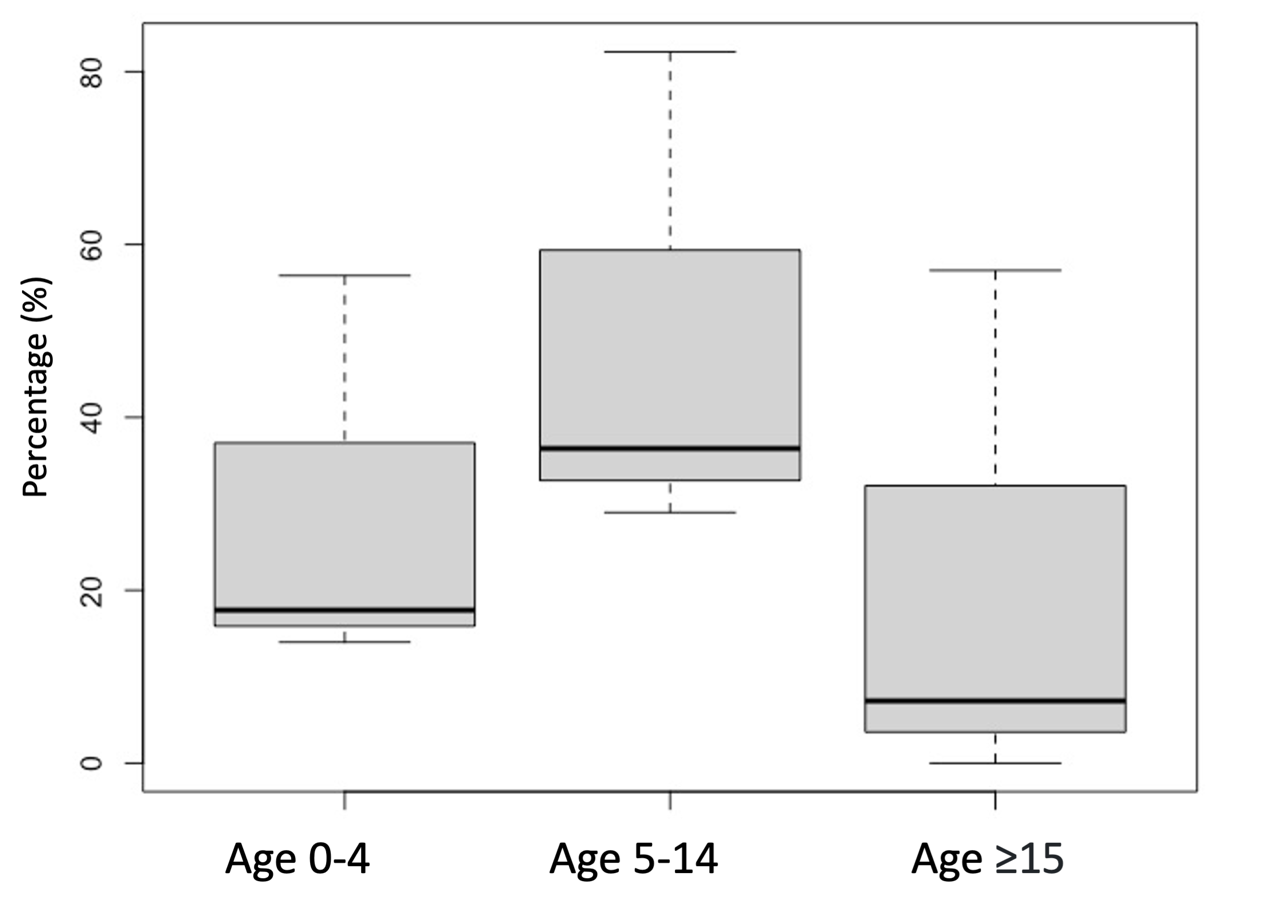


Supplementary Figure 3. Age distribution of cases among only culture-confirmed cases. Boxes, horizontal black lines, whiskers, and dots represent IQR (interquartile range), median, 1.5 IQR, and outliers, respectively.

A meta-analysis of the attack rate and case fatality rate (CFR) of the typhoid outbreaks was performed. Subgroup analysis was conducted by six WHO regions: African Region (AFR), Region of the Americas (AMR), Eastern Mediterranean Region (EMR), European Region (EUR), South-East Asia Region (SEAR), and Western Pacific Region (WPR).

Attack Rate

The pooled estimate of the attack rate was 0.98% (95% confidence interval, 0.37% - 2.59%) based on data from 20 outbreaks. The attack rate in the South-East Asia Region (SEAR) was the highest, with an estimated proportion of 3.60% (95% CI, 1.59% - 7.94%), followed by the African Region (AFR) with 0.5% (95% CI, 0.22% - 1.14%), and Eastern Mediterranean Region (EMR) with 0.14% (95% CI, 0.00% - 6.06%). Only one outbreak reported an attack rate in the European Region (EUR) and the Western Pacific Region (WPR) (Supplementary Figure 4).


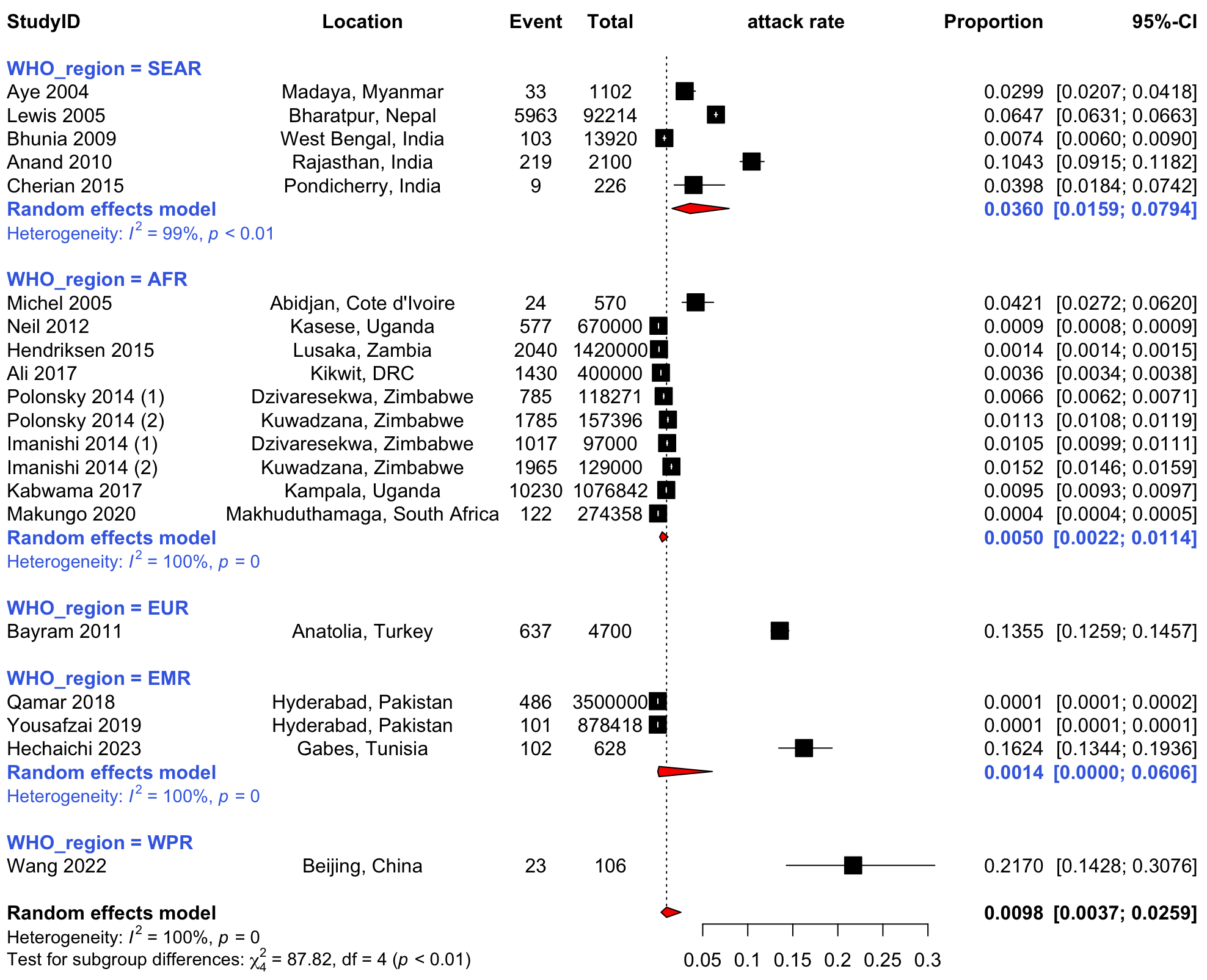


Supplementary Figure 4. Forest plot of attack rate

**Case Fatality Rate (CFR)**

The case fatality rate (CFR) was estimated based on the total number of patients who died (sum of suspected, probable, and confirmed cases or suspected cases if it includes other categories). The pooled estimate of CFR by total patients was 0.53% (95% CI, 0.17% - 1.65%). The CFR was highest in followed by African Region (AFR), estimated at 0.61% (95% CI, 0.14% - 2.50%), followed by the Eastern Mediterranean Region (EMR) at 0.34% (95% CI, 0.09% - 1.35%) (Supplementary Figure 5).


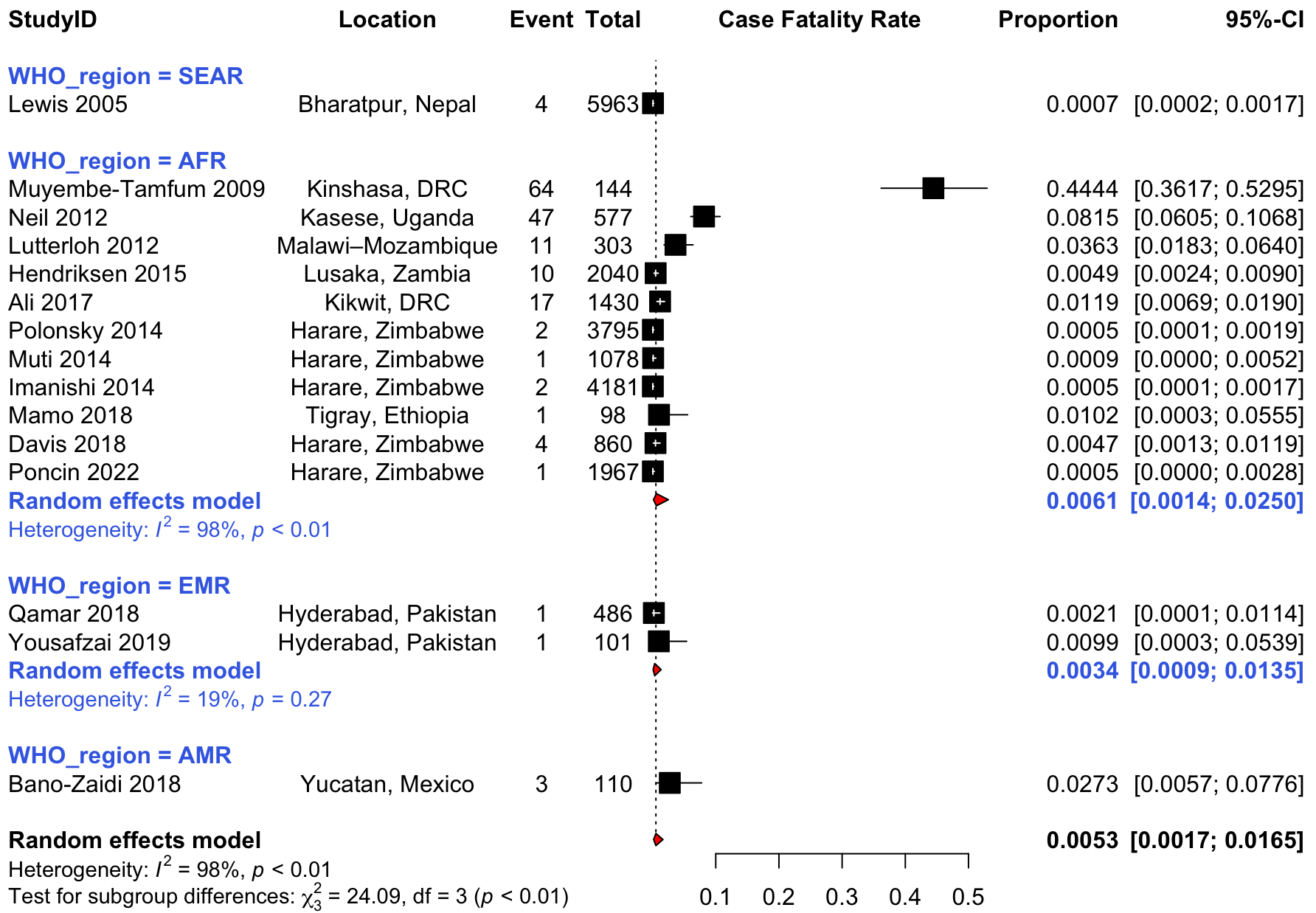


Supplementary Figure 5. Forest plot of CFR by total patients

### Reference

1 Al-Sanouri, T. M. *et al.* Emergence of plasmid-mediated multidrug resistance in epidemic and non-epidemic strains of Salmonella enterica serotype Typhi from Jordan. *J Infect Dev Ctries* **2**, 295-301 (2008). <https://doi.org:10.3855/jidc.225>

2 Ali, E. *et al.* Localised transmission hotspots of a typhoid fever outbreak in the Democratic Republic of Congo. *Pan Afr Med J* **28**, 179 (2017). <https://doi.org:10.11604/pamj.2017.28.179.10208>

3 Aye, T. T. & Siriarayapon, P. Typhoid fever outbreak in Madaya Township, Mandalay Division, Myanmar, September 2000. *J Med Assoc Thai* **87**, 395-399 (2004).

4 Bano-Zaidi, M. *et al.* Typhoid fever outbreak with severe complications in Yucatan, Mexico. *Lancet Glob Health* **6**, e1062-e1063 (2018). <https://doi.org:10.1016/S2214-109X(18)30312-7>

5 Bayram, Y. *et al.* Epidemiological characteristics and molecular typing of Salmonella enterica serovar Typhi during a waterborne outbreak in Eastern Anatolia. *Ann Trop Med Parasitol* **105**, 359-365 (2011). <https://doi.org:10.1179/1364859411Y.0000000024>

6 Burnsed, L. J. *et al.* Use of whole genome sequencing to complement characterisation of a typhoid fever outbreak among a Marshallese community: Oklahoma, 2015. *Epidemiol Infect* **147**, e11 (2018). <https://doi.org:10.1017/S0950268818002601>

7 Cherian, J. *et al.* An outbreak investigation of typhoid fever in Pondicherry, South India, 2013. *Int J Med Sci Public Health* **4**, 256-261 (2015).

8 Davis, W. W. *et al.* Notes from the Field: Typhoid Fever Outbreak - Harare, Zimbabwe, October 2016-March 2017. *MMWR Morb Mortal Wkly Rep* **67**, 342-343 (2018). <https://doi.org:10.15585/mmwr.mm6711a7>

9 Hechaichi, A. *et al.* Outbreak Investigation of Typhoid Fever in the District of Gabes, South of Tunisia. *Epidemiologia (Basel)* **4**, 223-234 (2023). <https://doi.org:10.3390/epidemiologia4030023>

10 Hendriksen, R. S. *et al.* Genomic signature of multidrug-resistant Salmonella enterica serovar typhi isolates related to a massive outbreak in Zambia between 2010 and 2012. *J Clin Microbiol* **53**, 262-272 (2015). <https://doi.org:10.1128/JCM.02026-14>

11 Holt, K. E. *et al.* Temporal fluctuation of multidrug resistant salmonella typhi haplotypes in the mekong river delta region of Vietnam. *PLoS Negl Trop Dis* **5**, e929 (2011). <https://doi.org:10.1371/journal.pntd.0000929>

12 Hu, B. *et al.* Genomic Investigation Reveals a Community Typhoid Outbreak Caused by Contaminated Drinking Water in China, 2016. *Front Med (Lausanne)* **9**, 753085 (2022). <https://doi.org:10.3389/fmed.2022.753085>

13 Imanishi, M. *et al.* Household water treatment uptake during a public health response to a large typhoid fever outbreak in Harare, Zimbabwe. *Am J Trop Med Hyg* **90**, 945-954 (2014). <https://doi.org:10.4269/ajtmh.13-0497>

14 Kabwama, S. N. *et al.* A large and persistent outbreak of typhoid fever caused by consuming contaminated water and street-vended beverages: Kampala, Uganda, January - June 2015. *BMC Public Health* **17**, 23 (2017). <https://doi.org:10.1186/s12889-016-4002-0>

15 Keddy, K. H. *et al.* Molecular epidemiological investigation of a typhoid fever outbreak in South Africa, 2005: the relationship to a previous epidemic in 1993. *Epidemiol Infect* **139**, 1239-1245 (2011). <https://doi.org:10.1017/S0950268810002207>

16 Lewis, M. D. *et al.* Typhoid fever: a massive, single-point source, multidrug-resistant outbreak in Nepal. *Clin Infect Dis* **40**, 554-561 (2005). <https://doi.org:10.1086/427503>

17 Limpitikul, W., Henpraserttae, N., Saksawad, R. & Laoprasopwattana, K. Typhoid outbreak in Songkhla, Thailand 2009-2011: clinical outcomes, susceptibility patterns, and reliability of serology tests. *PLoS One* **9**, e111768 (2014). <https://doi.org:10.1371/journal.pone.0111768>

18 Lutterloh, E. *et al.* Multidrug-resistant typhoid fever with neurologic findings on the Malawi-Mozambique border. *Clin Infect Dis* **54**, 1100-1106 (2012). <https://doi.org:10.1093/cid/cis012>

19 Makungo, U. B. *et al.* Epidemiological investigation of a typhoid fever outbreak in Sekhukhune District, Limpopo province, South Africa - 2017. *S Afr J Infect Dis* **35**, 107 (2020). <https://doi.org:10.4102/sajid.v35i1.107>

20 Michel, R. *et al.* Outbreak of typhoid fever in vaccinated members of the French Armed Forces in the Ivory Coast. *Eur J Epidemiol* **20**, 635-642 (2005). <https://doi.org:10.1007/s10654-005-7454-6>

21 Muehlen, M. *et al.* Outbreak of domestically acquired typhoid fever in Leipzig, Germany, June 2004. *Eurosurveillance* **12**, 7-8 (2007). <https://doi.org:10.2807/ESM.12.02.00684-EN>

22 Muti, M. *et al.* Typhoid outbreak investigation in Dzivaresekwa, suburb of Harare City, Zimbabwe, 2011. *Pan Afr Med J* **18**, 309 (2014). <https://doi.org:10.11604/pamj.2014.18.309.4288>

23 Muyembe-Tamfum, J. J. *et al.* An outbreak of peritonitis caused by multidrug-resistant Salmonella Typhi in Kinshasa, Democratic Republic of Congo. *Travel Med Infect Dis* **7**, 40-43 (2009). <https://doi.org:10.1016/j.tmaid.2008.12.006>

24 N'Cho H, S. *et al.* Notes from the Field: Typhoid Fever Outbreak - Harare, Zimbabwe, October 2017-February 2018. *MMWR Morb Mortal Wkly Rep* **68**, 44-45 (2019). <https://doi.org:10.15585/mmwr.mm6802a5>

25 Neil, K. P. *et al.* A large outbreak of typhoid fever associated with a high rate of intestinal perforation in Kasese District, Uganda, 2008-2009. *Clin Infect Dis* **54**, 1091-1099 (2012). <https://doi.org:10.1093/cid/cis025>

26 Nimonkar, R. A., Goyal, A. K., Ahmed, S., Pardal, M. P. S. & Singh, S. Clinico-epidemiological study of an outbreak of typhoid in North India. *J Family Med Prim Care* **11**, 3570-3574 (2022). <https://doi.org:10.4103/jfmpc.jfmpc_2498_21>

27 Polonsky, J. A. *et al.* Descriptive epidemiology of typhoid fever during an epidemic in Harare, Zimbabwe, 2012. *PLoS One* **9**, e114702 (2014). <https://doi.org:10.1371/journal.pone.0114702>

28 Poncin, M. *et al.* Implementation of an outbreak response vaccination campaign with typhoid conjugate vaccine - Harare, Zimbabwe, 2019. *Vaccine X* **12**, 100201 (2022). <https://doi.org:10.1016/j.jvacx.2022.100201>

29 Qamar, F. N. *et al.* Outbreak investigation of ceftriaxone-resistant Salmonella enterica serotype Typhi and its risk factors among the general population in Hyderabad, Pakistan: a matched case-control study. *Lancet Infect Dis* **18**, 1368-1376 (2018). <https://doi.org:10.1016/S1473-3099(18)30483-3>

30 Roy, J. S., Saikia, L., Medhi, M. & Tassa, D. Epidemiological investigation of an outbreak of typhoid fever in Jorhat town of Assam, India. *Indian J Med Res* **144**, 592-596 (2016). <https://doi.org:10.4103/0971-5916.200902>

31 Scobie, H. M. *et al.* Impact of a targeted typhoid vaccination campaign following cyclone Tomas, Republic of Fiji, 2010. *Am J Trop Med Hyg* **90**, 1031-1038 (2014). <https://doi.org:10.4269/ajtmh.13-0728>

32 Srinivasan, M. *et al.* Outbreak of Typhoid Fever in Children of Urban Vellore: A Report from the Surveillance for Enteric Fever in India Cohort. *Am J Trop Med Hyg* **107**, 82-85 (2022). <https://doi.org:10.4269/ajtmh.21-0593>

33 Walters, M. S. *et al.* Shifts in geographic distribution and antimicrobial resistance during a prolonged typhoid fever outbreak--Bundibugyo and Kasese Districts, Uganda, 2009-2011. *PLoS Negl Trop Dis* **8**, e2726 (2014). <https://doi.org:10.1371/journal.pntd.0002726>

34 Wang, Y. *et al.* Extensively Drug-Resistant (XDR) Salmonella Typhi Outbreak by Waterborne Infection - Beijing Municipality, China, January-February 2022. *China CDC Wkly* **4**, 254-258 (2022). <https://doi.org:10.46234/ccdcw2022.062>

35 Yousafzai, M. T. *et al.* Ceftriaxone-resistant Salmonella Typhi Outbreak in Hyderabad City of Sindh, Pakistan: High Time for the Introduction of Typhoid Conjugate Vaccine. *Clin Infect Dis* **68**, S16-S21 (2019). <https://doi.org:10.1093/cid/ciy877>
